# Development and validation of the Chinese coaches’ interpersonal style scale

**DOI:** 10.3389/fpsyg.2023.1290549

**Published:** 2023-12-13

**Authors:** Wen Su, Daliang Zhao

**Affiliations:** ^1^Public Courses Teaching Department, Guangzhou Sport University, Guangzhou, China; ^2^College of Leisure and Digital Sports, Guangzhou Sport University, Guangzhou, China

**Keywords:** coach, interpersonal style, benevolent style, autonomy-supportive style, controlling style

## Abstract

**Purpose:**

Coaches’ behaviors and coaching styles play a critical role in influencing athletes’ psychological experiences and performance. According to the self-determination theory (SDT), coaches’ interpersonal behaviors are commonly categorized as autonomy-supportive and controlling. Due to less focus on the unique behaviors of Chinese coaches, this study incorporated coaches’ parental care for athletes, referred to as paternalistic benevolence, in their interpersonal styles in the context of the Chinese culture.

**Methods:**

Exploratory factor analyses were used in studies 1 and 2 to find items associated with benevolent coaching behaviors and items to create the Chinese Coaches’ Interpersonal Style Scale. Study 3 used the constructed scale, as well as the Subjective Vitality Scale and Athlete Burnout Questionnaire, with a sample of athletes to examine scale reliability. The 15-item Chinese Coaches’ Interpersonal Style Scale contained three dimensions: benevolent, autonomy-supportive, and controlling coaching styles.

**Results:**

The findings showed that: (1) benevolent coaching behaviors held significant explanatory weight in the Chinese cultural context; (2) controlling and autonomy-supportive coaching styles were culturally congruent among both Eastern and Western athletes; and (3) benevolent and autonomy-supportive coaching behaviors positively impacted athletes, whereas controlling coaching behaviors had a negative impact.

**Conclusion:**

The measure showed strong validity and reliability, making it useful for future practice and research on the interpersonal style of Chinese coaches.

## 1 Introduction

The psychological experiences and performance of players in sports are profoundly influenced by coaches’ behaviors (Lemelin et al., 2022). Coaches’ styles are generally classified into two categories based the self-determination theory (SDT): autonomy-supportive and controlling (Vallerand and Losier, 1999). Previous studies have consistently demonstrated that coaches’ interpersonal styles have a significant impact on athletes’ basic psychological need, motivation, and well-being in competitive sports (Mageau and Vallerand, 2003; Balaguer et al., 2012; Curran et al., 2014; Healy et al., 2014). Autonomy-supportive coaches promote freedom, encourage autonomy, and involve athletes in decision-making processes. Conversely, coaches with controlling styles demonstrate coercive, authoritarian, and pressure acts. Athletes’ perception of their coaches’ interpersonal styles predict changes in the psychological need satisfaction or thwarting, impacting their subjective vitality and burnout (Balaguer et al., 2012). In particular, perceiving an autonomy-supportive environment is positively correlated with subjective vitality and need satisfaction and negatively correlated with burnout and need thwarting (Stebbings et al., 2012; Amorose and Anderson-Butcher, 2015; Mossman et al., 2022). In contrast, perceiving a controlling environment is positively correlated with need thwarting and burnout and negatively correlated with subjective vitality (Bartholomew et al., 2011b; Amorose and Anderson-Butcher, 2015; González et al., 2017; Ntoumanis et al., 2017).

Several surveys have analyzed coaches’ interpersonal behavior. The short version of the Sport Climate Questionnaire (SCQ) has been used to examine players’ experiences with their coaches’ autonomy-supportive behavior (Amorose and Anderson-Butcher, 2007; Amorose et al., 2016). This six-item scale, derived from the Health Care Climate Questionnaire (Williams et al., 1998), assesses whether coaches support athletes’ psychological needs (Standage et al., 2006). Example items include “I feel that my coach provides us choices and options” and “I feel understood by my coach.” Moreover, the Autonomy-Supportive Coaching Questionnaire, developed by Conroy and Coatsworth (2007), examines autonomy support in two dimensions: interest in athlete’s input and praise for autonomous behavior. Other scales adapted from various domains include the Perceived Autonomy Support Scale for Exercise Setting (Gillet et al., 2010), Interpersonal Supportiveness Scale-Coach (Wilson et al., 2009), and Problem in Sports Questionnaire (Carpentier and Mageau, 2013). Conversely, the Controlling Coach Behavior Scale (CCBS) assesses negative features of coaching styles through four aspects: controlling use of rewards, negative conditioned regard, intimidation, and excessive personal control (Bartholomew et al., 2010). Numerous studies have evaluated coaches helping and hindering actions. The SDT and achievement goal theory serve as the theoretical foundation of the Empowering and Disempowering Motivational Climate Questionnaire-Coach, which includes the dimensions of task-involving, autonomy-supportive, socially-supportive, ego-involving, and controlling coaching. However, this questionnaire has several problematic items, despite having been tested with a variety of methodologies (Appleton et al., 2016). Furthermore, the Interpersonal Behaviors Questionnaire and Coaches’ Interpersonal Style Questionnaire examines basic psychological needs. Both of these scales have six components: autonomy support, autonomy thwarting, competence support, competence thwarting, relatedness support, and relatedness thwarting (Rocchi et al., 2017; Pulido et al., 2018).

Most existing coaching-style scales have been developed for Western cultural contexts. It is crucial to understand how cultural factors impact coaching behaviors in a range of cultural situations. Several cross-cultural studies have demonstrated that the SDT is applicable to athletes from various countries; however, pathway size and degree of variance explained in outcome variables vary (Jowett et al., 2017). For instance, the satisfaction of basic psychological needs was found to explain changes in autonomous motivation among the majority of Chinese athletes, a moderate number of Greek and Swedish athletes, and a small proportion of Spanish and British athletes (Jowett et al., 2017). Furthermore, the relatedness of psychological needs may vary across nations and cultures (Maulana et al., 2013). Focusing on autonomy may be beneficial in highly individualistic Western societies that emphasize autonomy (Oishi, 2000). In contrast, in cultures focused on authority, such as China and Greece, a lack of autonomy may not always be detrimental (Miller, 2014). In addition, self-determined motivation was found to have a stronger impact on Chinese and Greek athletes than on Spanish and British athletes (Jowett et al., 2017). This cross-cultural disparity is considerable in both collectivist (e.g., China) and individualist societies (Hofstede and Hofstede, 2001; Maulana et al., 2013).

Moreover, perceptions of the coach-athlete relationship differ, with Western athletes perceiving it as a partnership, and Chinese athletes often equating it to a parent–child relationship. Moreover, under the supervision of their coaches, Chinese athletes have limited control over their private lives, whereas Western athletes have greater personal freedom and autonomy (Li et al., 2015). An old Chinese proverb, “A day as a teacher, a lifetime as a parent,” implies that even a teacher who imparts knowledge for only one day should be treated as a lifetime parent. This is especially essential for Chinese athletes, many of whom begin their athletic careers at an early age. Therefore, coaches in China not only offer their expertise but also play a parental role in athletes’ lives. Paternalistic benevolence has emerged as a distinguishing trait of coaches’ parental responsibilities in the coach-athlete relationship (Farh et al., 2000). Benevolence is defined by an explicit distinction between superior and subordinate roles, in which the superior accepts the obligation to care for the inferior, who reciprocates with appreciation, loyalty, and obedience. Benevolence is effective in contexts with a significant gap between superiors and subordinates, whereas autonomy support thrives in an egalitarian environment (Farh et al., 2000). Benevolence extends beyond athletic expertise in the coach-athlete relationship to encompass the personal care and protection of athletes.

The universal applicability of the SDT across cultures has been established, with coaching styles playing a critical role in promoting athletic well-being worldwide (Jowett et al., 2017). The influence of an autonomy-supportive coaching style on athletes is consistent and independent of culture and sport type (Mossman et al., 2022). Efforts have also been made to adapt the CCBS to the Chinese culture, with findings indicating that dimensions such as excessive personal control and negative conditioned regard retain cross-cultural congruence across Eastern and Western athletes (Zhao and Zhou, 2022). Hence, this study aimed to establish and validate a scale to examine coaches’ interpersonal styles in the Chinese cultural context.

## 2 Study 1

### 2.1 Methods

#### 2.1.1 Participants

The participants (*N* = 148) comprised 77 men and 71 women aged 13–30 years (*M* = 20, *SD* = 3.079), including age groups of 13–15 (*n* = 8), 16–20 (*n* = 87), 21–25 (*n* = 47), and 26–30 years (*n* = 6). Their training experience was 0–23 years (*M* = 6.66, *SD* = 4.033), with training periods including 0–5 (*n* = 75), 6–10 (*n* = 49), 11–15 (*n* = 20), 16–20 (*n* = 3), and 21–23 years (*n* = 1). The athletes participated in three sports: athletics (*n* = 100), martial arts (*n* = 39), and gymnastics (*n* = 9). All procedures were approved by the Institutional Review Board of Guangzhou Sport University. All participants or their parents provided written informed consent forms.

#### 2.1.2 Measures

We used the benevolent leadership subscale of the Paternalistic Leadership Scale (PLS; Farh et al., 2000). This subscale consisted of 11 items distributed across two dimensions: individual care, which included six items (e.g., “The leader expresses concern about my daily life”), and understanding and forgiveness, which included five items (e.g., “The leader encourages me when I encounter arduous problems”). Responses were rated on a five-point Likert scale (1 = never; 5 = always).

We modified the PLS by transferring it from an enterprise leadership context to a sports environment. During the revision phase, we improved the benevolence dimension by deleting three items that were irrelevant to the Chinese sports context. Benevolence yielded a final set of eight items.

#### 2.1.3 Data analysis

Data analysis was conducted using SPSS 20.0, and each item was examined using an exploratory factor analysis (EFA). Items with factor loadings greater than 0.40 were considered acceptable (Guadagnoli and Velicer, 1988; Samuels, 2017). Items with factor loadings less than 0.4 and significant cross-loadings (two or more factor loadings more than 0.40) were excluded (Ferguson and Cox, 1993).

### 2.2 Results

#### 2.2.1 Exploratory factor analysis

The EFA and extraction used the principal component analysis and identified two co-factors. Varimax rotation was used to examine benevolent coaching behaviors. The sample suitability test (Kaiser-Meyer-Olkin, KMO = 0.86) and spherical test (*χ*^2^ = 620.29, *p* < 0.001) revealed that the sample was adequate for factor analysis. All items had factor loadings greater than 0.40, and one item in the individual care (λ = 0.62) had a cross-loading greater than 0.40 (λ = 0.44) in the understanding and forgiveness (e.g., “My coach often shows concern about me”). Moreover, in the understanding and forgiveness dimension, one item (λ = 0.55) had a greater cross-loading (λ = 0.63) for individual care (e.g., “My coach encourages me when I encounter arduous problems”). Based on the principal component loadings, these two items were eliminated in turn.

Subsequently, each factor had an eigenvalue greater than 1, and the cumulative contribution accounted for 73.52% of the total variance. The eigenvalue of individual care was 2.25, explaining 37.49% of the interpretable variance, whereas the eigenvalue of understanding and forgiveness was 2.16, explaining 36.04% of the interpretable variance. Item factor loadings varied from 0.79 to 0.87. Thus, benevolent coaching style was divided into two dimensions (individual care and understanding and forgiveness), with three items each (Table 1).

**Table 1:** Descriptive statistics and factor loadings based on the exploratory factor analysis (Study 1).

Item	*M*	*SD*	Skewness	Kurtosis	F1	F2
Individual care						
1. Beyond training, my coach expresses concern about my daily life	3.97	0.94	−0.38	−0.77	**0.79**	0.30
3. My coach meets my needs according to my personal requests	3.67	0.92	−0.09	−0.38	**0.84**	0.18
4. My coach handles what is difficult to do or manage in everyday life for me	3.84	0.94	−0.37	−0.34	**0.87**	0.22
Understanding and forgiveness						
6. My coach tries to understand the cause if I do not perform well	4.35	0.76	−0.79	−0.53	0.27	**0.85**
7. When I make mistakes, my coach gives me the opportunity to make amends	4.41	0.65	−0.65	−0.57	0.29	**0.81**
8. My coach avoids embarrassing me in front of my teammates	3.78	1.02	−0.33	−0.71	0.14	**0.79**

Items were modified from the Paternalistic Leadership Scale (Farh et al., 2000). F1 = Individual care, F2 = Understanding and forgiveness. The primary factor loadings are in bold.

## 3 Study 2

### 3.1 Methods

#### 3.1.1 Participants

A total of 241 athletes from Guangdong Province participated in Study 2, including 132 men and 109 women aged 11–30 years (*M* = 18.76, *SD* = 3.700), with age groups including 11–15 (*n* = 43), 16–20 (*n* = 125), 21–25 (*n* = 61), and 26–30 years (*n* = 12). Their training experience was 1–20 years (*M* = 8.49, *SD* = 4.024), with training periods including 1–5 (*n* = 61), 6–10 (*n* = 113), 11–15 (*n* = 55), and 16–20 years (*n* = 12). The athletes were engaged in various sports: fencing (*n* = 49), weightlifting (*n* = 28), badminton (*n* = 27), water polo (*n* = 26), swimming (*n* = 24), athletics (*n* = 17), gymnastics (*n* = 17), artistic swimming (*n* = 17), table tennis (*n* = 15), sanda (*n* = 10), tennis (*n* = 6), and Wushu (*n* = 5). All procedures were approved by the Institutional Review Board of Guangzhou Sport University. All participants or their parents provided written informed consent forms.

#### 3.1.2 Measures

The Benevolent coaching style measure developed in Study 1 was used. The scale contained two dimensions: individual care, which contained three items (e.g., “Beyond training, my coach expresses concern about my daily life”), and understanding and forgiveness, which comprised three items (e.g., “My coach tries to understand the cause if I do not perform well”). Responses were rated on a seven-point Likert scale (1 strongly disagree; 7 = strongly agree).

In addition, we used the six-item SCQ to assess athletes’ perceived autonomy support of coaches. This questionnaire was originally designed for the health domain but was later modified for the sports domain (Reinboth et al., 2004), with items such as “I feel that my coach provides me choices and options.” The redesigned measure demonstrated good psychometric properties in a sample of young athletes (Reinboth et al., 2004). Responses were rated on a seven-point Likert scale (1 = strongly disagree; 7 = strongly agree).

The CCBS is a self-report scale based on the SDT (Ryan and Deci, 2002) and developed to evaluate coaches’ controlling behaviors (Bartholomew et al., 2010). This scale consists of four factors: controlling the use of rewards, negative conditioned regard, intimidation, and excessive personal control. Previous studies using linear mixed models found that the perception of autonomy-supportive coaching behaviors were associated with basic need satisfaction and well-being, whereas controlling coaching behaviors (negative conditioned regard and excessive personal control) were associated with basic need frustration and poor well-being (Cheval et al., 2017). Therefore, these two factors are significant predictors of athletes’ well-being. The CCBS has been modified for the Chinese culture. Studies have shown that Eastern and Western athletes shared similar experiences of negative conditioned regard and excessive personal control (Zhao and Zhou, 2022). Therefore, Study 2 used the CCBS designed for Chinese athletes, which consisted of six items distributed across two dimensions (Zhao and Zhou, 2022). Negative conditioned regard included three items (e.g., “My coach is less supportive of me when I am not training and completing well”), and excessive personal control included three items (e.g., “My coach tries to control what I do in my free time”). Responses were rated on a seven-point Likert scale (1 = strongly disagree; 7 = strongly agree).

#### 3.1.3 Data analysis

SPSS 20.0 was used to analyze the data, and EFA was used to evaluate each item. Items with factor loadings less than 0.4 and high cross-loadings were eliminated (Guadagnoli and Velicer, 1988; Ferguson and Cox, 1993).

### 3.2 Results

#### 3.2.1 Exploratory factor analysis

We used the EFA with principal component analysis to identify three cofactors, followed by varimax rotation. The sample’s fitness for factor analysis was validated using the sample suitability test (KMO = 0.91) and spherical test (*χ*^2^ = 3241.98, *p* < 0.001). All items showed factor loadings above 0.40, except for one item (“My coach tries to understand the cause if I do not perform well”) in the benevolent coaching style dimension (λ = 0.65), which also displayed a cross-loading exceeding 0.40 in autonomy-supportive coaching style (λ = 0.41). Therefore, this item was excluded from the analysis.

Subsequently, the eigenvalues of the obtained factors were greater than 1, resulting in a cumulative contribution of 68.65%. In particular, the eigenvalue of benevolent coaching style was 2.92, which accounted for 17.17% of the interpretable variance. Autonomy-supportive coaching style had an eigenvalue of 4.65 and explained 27.33% of the interpretable variance. The eigenvalue of controlling coaching style was 4.11, and the explained variance was 24.15%. Items in these three factors had factor loadings ranging from 0.55 to 0.87. Thus, benevolent, autonomy-supportive, and controlling coaching styles were included as three components in the Chinese Coaches’ Interpersonal Style Scale (CCISS), with five, six, and six items, respectively (Table 2).

**Table 2:** Descriptive statistics and factor loadings based on the exploratory factor analysis (Study 2).

Item	*M*	*SD*	Skewness	Kurtosis	F1	F2	F3
Benevolent coaching style							
1. Beyond training, my coach expresses concern about my daily life	4.82	1.39	−0.09	−0.53	**0.72**	0.29	0.07
3. My coach meets my needs according to my personal requests	4.50	1.38	0.03	−0.07	**0.80**	0.20	0.03
4. My coach handles what is difficult to do or manage in everyday life for me	4.93	1.46	−0.29	−0.45	**0.76**	0.37	−0.10
7. When I make mistakes, my coach gives me the opportunity to make amends	4.50	1.53	−0.11	−0.51	**0.55**	0.25	−0.32
8. My coach avoids embarrassing me in front of my teammates	5.54	1.15	−0.62	−0.04	**0.61**	0.27	−0.33
Autonomy-supportive coaching style							
9. I feel that my coach provides us choices and options	5.20	1.29	−0.32	−0.47	0.29	**0.77**	−0.15
10. I feel understood by my coach	4.92	1.43	−0.34	−0.31	0.24	**0.85**	−0.10
11. My coach conveyed confidence in my ability to do well at athletics	5.20	1.25	−0.26	−0.41	0.32	**0.84**	−0.22
12. My coach encouraged me to ask questions	5.52	1.25	−0.46	−0.57	0.30	**0.74**	−0.20
13. My coach listens me to how I would like to do things	5.18	1.40	−0.35	−0.62	0.26	**0.87**	−0.23
14. My coach tries to understand how I see things before suggesting a new way to do things	4.94	1.45	−0.35	−0.25	0.29	**0.80**	−0.21
Controlling coaching style							
15. My coach is less supportive of me when I am not training and competing well	3.07	1.54	0.26	−0.71	−0.13	−0.36	**0.69**
16. My coach pays me less attention if I have displeased him/her	3.33	1.52	0.21	−0.53	−0.03	−0.32	**0.71**
17. My coach is less accepting of me if I have disappointed him/her	3.48	1.54	0.11	−0.63	−0.07	−0.22	**0.78**
18. My coach tries to control what I do in my free time	2.75	1.50	0.80	0.24	−0.04	−0.08	**0.84**
19. My coach tries to interfere in aspects of my life outside of my sport	2.71	1.37	0.64	0.17	−0.11	−0.08	**0.82**
20. My coach tries to control everything I did	2.50	1.35	0.92	0.71	−0.09	0.00	**0.84**

Items of the Autonomy-supportive coaching style were derived from the Sport Climate Questionnaire (Reinboth et al., 2004). Items of the Controlling coaching style were derived from the Controlling Coach Behavior Scale (Bartholomew et al., 2010; Zhao and Zhou, 2022). F1 = Benevolent, F2 = Autonomy-supportive, F3 = Controlling. The primary factor loadings are in bold.

## 4 Study 3

We conducted a confirmatory factor analysis (CFA) of the CCISS to determine the suitability of the three-dimensional division based on Schumann’s seven-point guide (Schumann et al., 2022).

### 4.1 Methods

#### 4.1.1 Participants

A total of 531 athletes from Guangdong Province, including 268 men and 263 women, participated in Study 3. The participants’ ages ranged from 10 to 31 years (*M* = 18.68, *SD* = 3.973), with the age groups including 10–15 (*n* = 103), 16–20 (*n* = 275), 21–25 (*n* = 127), and 26–31 years (*n* = 26). The participants’ training periods were 1–26 years (*M* = 7.98, *SD* = 4.155), including ranges of 1–5 (*n* = 172), 6–10 (*n* = 232), 11–15 (*n* = 107), 16–20 (*n* = 17), and 21–26 years (*n* = 3). The sports represented covered a diverse range: athletics (*n* = 86), volleyball (*n* = 47), fencing (*n* = 46), gymnastics (*n* = 42), basketball (*n* = 41), trampolining (*n* = 39), water polo (*n* = 35), swimming (*n* = 33), table tennis (*n* = 30), weightlifting (*n* = 27), badminton (*n* = 25), artistic swimming (*n* = 21), diving (*n* = 21), Wushu (*n* = 21), sanda (*n* = 14), and tennis (*n* = 3). All procedures were approved by the Institutional Review Board of Guangzhou Sport University. All participants or their parents provided written informed consent forms.

#### 4.1.2 Measures

Based on the results of Study 2, the CCISS, which consists of 15 items divided into three coaching styles, was created. Benevolent coaching style was divided into two dimensions: individual care, which included three items (e.g., “Beyond training, my coach expresses concern about my daily life”), and understanding and forgiveness, which included two items (e.g., “My coach avoids embarrassing me in front of my teammates”). Understanding and forgiveness was eliminated from further analyses, as it contained only two items (Suhr, 2006). Thus, benevolent coaching style contained three items. Autonomy-supportive coaching style comprised six items, such as “I feel that my coach provides us choices and options.” Controlling coaching style included six items in two dimensions: negative conditioned regard, which included three items (e.g., “My coach is less supportive of me when I am not training and completing well”), and excessive personal control, which included three items (e.g., “My coach tries to control what I do in my free time”). The mean values of negative conditioned regard and excessive personal control were used as observation variables of controlling coaching style in the CFA. Responses were rated on a seven-point Likert scale (1 = strongly disagree; 7 = strongly agree).

The seven-item Subjective Vitality Scale Ryan and Frederick (1997) evaluates individuals’ perceptions of their vitality (e.g., “I feel alive and vital right now”). Responses were rated on a seven-point Likert scale (1 = not at all true; 7 = very true). The Chinese version of the scale exhibited an internal consistency of 0.87 and was found reliable in the context of exercise (Liu and Chung, 2014).

The 15-item Athlete Burnout Questionnaire was developed to evaluate athlete burnout (Raedeke and Smith, 2001), with three factors: reduced sense of accomplishment (5 items; e.g., “I am not achieving much in sports”), emotional or physical exhaustion (5 items; e.g., “I feel so tied from my training that I have trouble finding energy to do other things”), and devaluation (5 items; e.g., “The effort I spend on sports would be better spent doing other things”). Responses were rated on a five-point Likert scale (1 = almost never; 5 = almost always). Research has supported the reliability (Lemyre et al., 2006), structural validity (Raedeke and Smith, 2001), and convergent and discriminant validity (Cresswell and Eklund, 2006) of the scale.

#### 4.1.3 Data analysis

SPSS 20.0 and AMOS 28.0 were used for data analysis. The CFA was used to assess the structural validity of the CCISS. We utilized *χ*^2^/df, comparative fit index (CFI), Tucker-Lewis index (TLI), parsimony normative fit index (PNFI), and root mean square error of approximation (RMSEA) as model fit indices. The criteria for excellent fit are CFI ≥ 0.95, TLI ≥ 0.95, and RMSEA ≤0.06 (Hu and Bentler, 1999). Acceptable fit is indicated by CFI ≥ 0.90, TLI ≥ 0.90, RMSEA ≤0.080 (Browne and Cudeck, 1992; Hu and Bentler, 1999), and PNFI ≥0.60 (Netemeyer et al., 1990). In addition, for a larger sample size, the *χ*^2^/df should ideally be ≤4, with a lower index suggesting a better model fit (Hotchkiss and Cook-Cottone, 2019). A correlation analysis was used to test the validity of the results.

### 4.2 Results

#### 4.2.1 Confirmatory factor analysis

**Figure 1:**
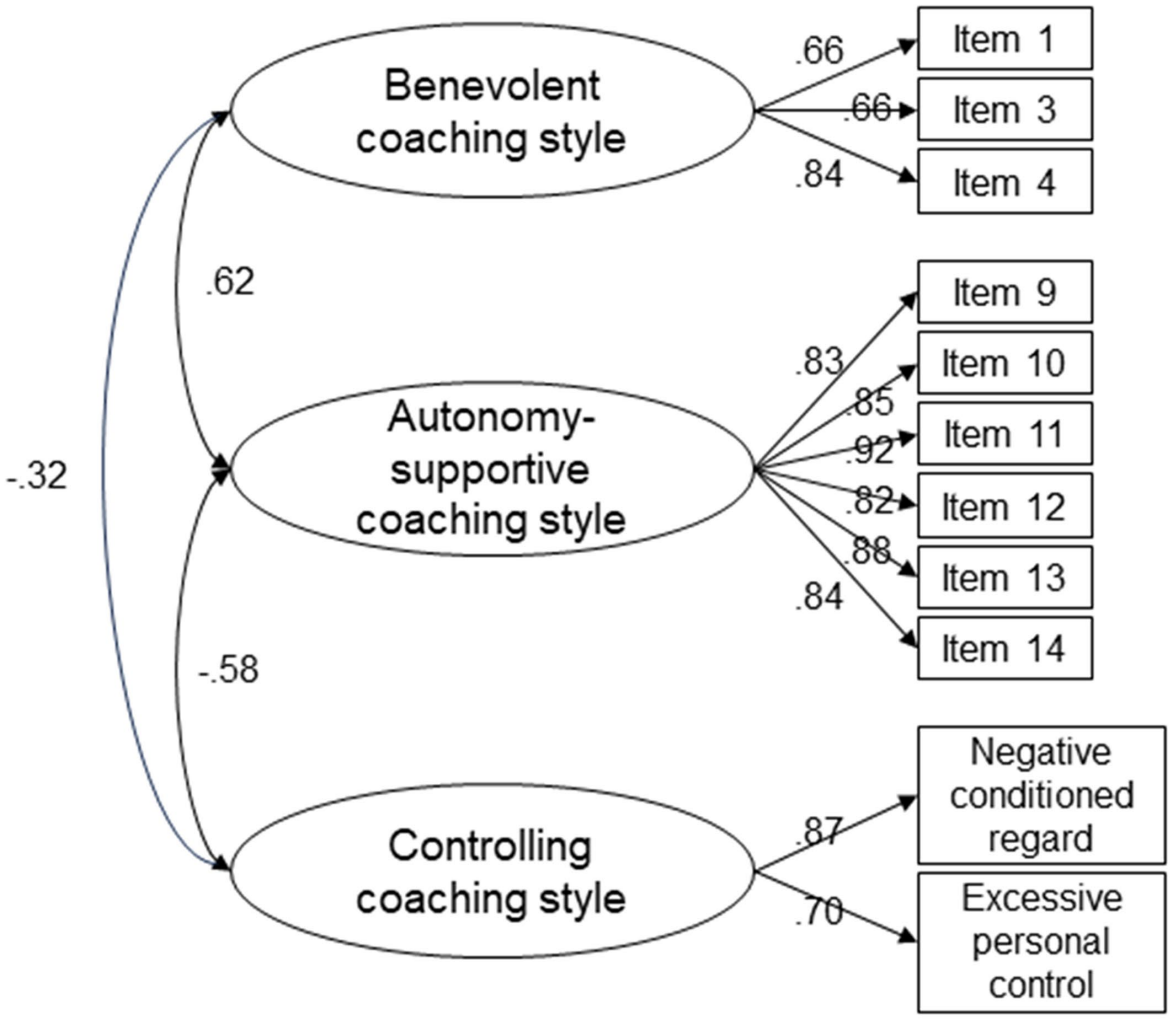
Results of the 3-factor confirmatory factor analysis of the Chinese coaches’ interpersonal style scale.

The CFA revealed a relatively good fit to the data, with room for some improvement: *χ*^2^/df = 6.004, RMSEA =0.097, CFI = 0.949, TLI =0.931, and PNFI = 0.700. Larger modification indices indicate possible residual correlations among certain items. Two rounds of residual correlations were performed. Items 13 and 14, belonging to autonomy-supportive coaching style, were the subject of the first modification, whereas items 12 and 13 were the focus of the second. The final model, which included 15 items in three dimensions (benevolent coaching style, three items; autonomy-supportive coaching style, six items; and controlling coaching style, six items), produced a substantially better fit to the data: *χ*^2^/df = 3.430, RMSEA = 0.068, CFI = 0.976, TLI = 0.967, and PNFI = 0.686 (Figure 1).

#### 4.2.2 Structural stability

Separate CFA was conducted for male and female participants to evaluate the stability of the CCISS structure. For female participants, three latent variables representing benevolent, autonomy-supportive, and controlling coaching styles were included. The results revealed the following fit indices: *χ*^2^/df = 3.470, RMSEA = 0.097, CFI = 0.951, TLI = 0.934, and PNFI = 0.696. Model fit indices after modification were: *χ*^2^/df = 2.695, RMSEA = 0.080, CFI = 0.968, TLI = 0.955, and PNFI = 0.674. For male participants, three latent variables indicating benevolent, autonomy-supportive, and controlling coaching styles were included. The results show the following fit indices: *χ*^2^/df = 4.377, RMSEA = 0.112, CFI = 0.930, TLI = 0.906, and PNFI = 0.680. The model fit indices improved after modifications: *χ*^2^/df = 2.575, RMSEA = 0.077, CFI = 0.969, TLI = 0.956, and PNFI = 0.674.

In addition, we divided sports into closed (e.g., athletics, gymnastics, trampolining, swimming, weightlifting, artistic swimming, diving, and Wushu) and open (e.g., volleyball, fencing, basketball, water polo, table tennis, badminton, sanda, and tennis) types. The CFA for the open sports were used as latent variables. The results showed the following fit indices: *χ*^2^/df = 3.652, RMSEA = 0.105, CFI = 0.945, TLI = 0.926, and PNFI = 0.690. After the modifications, the model fit indices improved to: *χ*^2^/df = 2.493, RMSEA = 0.079, CFI = 0.970, TLI = 0.958, and PNFI = 0.675. The three dimensions were used as latent variables in the closed sports. The results revealed the following fit indices: *χ*^2^/df = 4.012, RMSEA = 0.102, CFI = 0.941, TLI = 0.921, PNFI = 0.688. After adjustment, the model fit indices improved: *χ*^2^/df = 2.658, RMSEA = 0.076, CFI = 0.969, TLI = 0.956, and PNFI = 0.675.

#### 4.2.3 Correlation analysis

The results of the correlation analysis (Table 3) showed a strong relationship between coaching style and players’ subjective experience. Specifically, subjective vitality had a substantial negative relationship with controlling coaching style and a significant positive relationship with autonomy-supportive and benevolent coaching styles. Reduced sense of accomplishment was positively correlated with controlling coaching style and negatively correlated with autonomy-supportive and benevolent coaching styles. Furthermore, emotional or physical exhaustion and devaluation showed the same pattern as reduced sense of accomplishment.

**Table 3:** The convergent and divergent validity of the Chinese coaches’ interpersonal style scale.

	Benevolent			Autonomy-supportive			Controlling		
	Total	Sample 1	Sample 2	Total	Sample 1	Sample 2	Total	Sample 1	Sample 2
Subjective vitality	0.315^**^	0.319^**^	0.312^**^	0.505^**^	0.468^**^	0.534^**^	−0.322^**^	−0.267^**^	−0.366^**^
Reduced sense of accomplishment	−0.194^**^	−0.142^*^	−0.244^**^	−0.421^**^	−0.364^**^	−0.474^**^	0.344^**^	0.249^**^	0.431^**^
Emotional or physical exhaustion	−0.158^**^	−0.188^**^	−0.134^*^	−0.378^**^	−0.350^**^	−0.401^**^	0.400^**^	0.304^**^	0.461^**^
Devaluation	−0.192^**^	−0.202^**^	−0.185^**^	−0.408^**^	−0.361^**^	−0.444^**^	0.417^**^	0.305^**^	0.491^**^

^*^, ^**^p < 0.05, 0.01, respectively.

The 531 participants were randomly divided into two groups to test the stability of the divergent and convergent validity of the CCISS. Samples 1 and 2 comprised 266 and 265 participants, respectively. Both groups demonstrated the same relationship between coaching style and other factors (Table 3), indicating that the scale had a robust and stable level of divergent and convergent validity.

#### 4.2.4 Internal consistency

As an alternative method for evaluating the validity of the CCISS, the results showed adequate internal consistency for all three factors via Cronbach’s alpha, ranging from 0.761 to 0.944 (Table 4).

**Table 4:** Internal consistency of the Chinese coaches’ interpersonal style scale.

Benevolent	Autonomy-supportive	Controlling
0.761	0.944	0.885

## 5 Discussion

This study aimed to develop and evaluate a scale designed for Chinese coaches’ behaviors based on the SDT. Most frequent coaching styles were autonomy-supportive and controlled. The autonomy-supportive style exhibited cross-cultural consistency (Mossman et al., 2022). Meanwhile, the Chinese version of CCBS demonstrated that cultural moderation had no appreciable impact on negative conditioned regard and excessive personal control (Zhao and Zhou, 2022). Furthermore, our study included the benevolent factor in understanding of the influence of the special parent–child relationship between Chinese coach and athletes on coaching style. Items of the benevolent coaching style were eliminated after conducting the EFA in Studies 1 and 2. Moreover, Study 3 adopted the CFA to determine whether the benevolent, autonomy-supportive, and controlling coaching styles were consistent with the behaviors usually observed in Chinese coaches. Consequently, this study revised the CCISS for the Chinese cultural background. The final 15-item CCISS, which included benevolent (three items), autonomy-supportive (six items), and controlling (six items) coaching styles, demonstrated good reliability and validity.

Moreover, analyses of convergence, discrimination, stability, reproducibility, and generalizability indicated that the scale usage could be further expanded. The correlation analysis in Study 3 showed a consistent relationship between all samples and subjective vitality, reduced sense of accomplishment, emotional or physical exhaustion, and devaluation. Study 3 demonstrated robust stability, as the scale’s results remained steady regardless of the sample’s gender or the type of sport in which they engaged (open or closed sports). The participants were randomly divided into two groups. The findings showed that the relationship between the three coaching styles and the other variables was constant across all samples. Notable similarities in the overall patterns of reliability, correlations, and stability were evident across all samples.

Benevolent coaching style items were improved using the EFA in Studies 1 and 2. Items of benevolent leadership in the enterprise context were modified for the sports context, and items with high cross-loading were deleted. In line with the theory of high cross-loading (Ferguson and Cox, 1993), an item might contribute to individual care and understanding and forgiveness, limiting a clear distinction between two factors. Individual care is generally characterized by coaches’ paternal concern or considerateness for their athletes, whereas understanding and forgiveness is characterized by sensitive to players’ needs or opinions. The items “My coach often shows concern about me” and “My coach encourages me when I encounter arduous problems” did not adequately capture the distinction between individual care and understanding and forgiveness, resulting in a total of three items for each dimension. The CCISS was examined in Study 2. The item associated with understanding and forgiveness exhibited a high cross-loading in autonomy-supportive coaching style, making it difficult to separate different Chinese coaches’ behaviors, as it explained benevolent coaching style and overlapped with autonomy-supportive coaching style. The warmth, caring, and support provided by an autonomy-supportive coach encourages athletes to express themselves (Iachini, 2013; Gaudreau et al., 2016), which is akin to understanding and forgiveness. Benevolent leadership primarily manifests as individual care (Farh and Cheng, 2000). Furthermore, understanding and forgiveness was reduced to two items, falling short of the minimal criteria of three items with acceptable factor loadings and low cross-loadings (Samuels, 2017). Thus, understanding and forgiveness dimension was excluded from the analysis. Three items of individual care were retained in the benevolent coaching style dimension.

Our results were in line with previous studies (Amorose and Anderson-Butcher, 2007; Conroy and Coatsworth, 2007; Bartholomew et al., 2010; Stebbings et al., 2011; Zhao and Zhou, 2022), which found that controlling coaching style is positively correlated with negative affect and negatively correlated with positive affect. In contrast, autonomy-supportive coaching style has a positive relationship with positive affect and a negative relationship with negative affect. The basic psychological needs theory holds that people succeed when their basic psychological needs for relatedness are satisfied (Deci and Ryan, 2000), and the interpretation of these results is consistent with this theory. Consequently, subjective vitality and burnout are affected by the satisfaction or frustration of psychological needs, which have a significant mediating effect on the quality of athletes’ participation in sports (Bartholomew et al., 2011a; Balaguer et al., 2012; González et al., 2017). In particular, the satisfaction and frustration with athletes’ basic psychological needs were significantly predicted by their perceptions of an autonomy-supportive environment. Moreover, needs satisfaction is a strong predictor of subjective vitality and athlete burnout. However, according to athletes’ perceptions of the controlling environment, need thwarting was positively associated with an increase in athlete burnout (Balaguer et al., 2012). Furthermore, our findings indicated a positive relationship between benevolent coaching style and positive affect, and a negative relationship existed with negative affect. Previous research has shown that benevolence improves athletes’ psychological capital and reduces burnout (Firebaugh, 1980). A study that examined college baseball players discovered that benevolent behavior was negatively correlated with athlete burnout (Tseng and Lun, 2008). In addition, people in benevolent contexts often have higher level of energy and vitality (Martela et al., 2016). This could be a result of benevolence in supporting athletes’ needs and inspiring them to express appreciation to the coach (Kao and Chen, 2006). From a practical standpoint, this study emphasizes the value of fostering an environment that is autonomy-supportive and benevolent while taking precautions to avoid a controlling environment. Coaching behavior is crucial for improving athletes’ perceived vitality and reducing burnout. Coaches must reduce control and foster a supportive and benevolent environment to boost athletes’ subjective vitality and reduce burnout.

A study of paternalistic leadership in Eastern commercial organizations produced the concept of the benevolent dimension (Farh et al., 2000). In contrast, transformational leadership is frequently mentioned in Western leadership theories (Brown and Keeping, 2005). Despite some similarities (Bedi, 2020), Western transformational leadership and Eastern paternalistic leadership, which developed in different cultural contexts, have certain distinctions. Both types of leadership exhibit individual care. However, transformational leadership focuses on individual considerations in the work environment. In Western cultures, subordinates perceive a leader’s involvement in their private lives as an invasion of privacy (Aycan, 2006). Conversely, paternalistic leadership, which is more common in Eastern cultures, extends individual concern to both work and private aspects of subordinates’ lives (Cheng et al., 2004; Erben and Güneşer, 2008; Chen et al., 2014). These discrepancies in coach-athlete relationships between Eastern and Western countries may be attributed to this cultural distinction. Western societies place a greater emphasis on individualism, and the gap between leaders and subordinates is smaller, encouraging an equal relationship between coaches and players. In contrast, the emphasis on collectivism in China creates a wider difference in power between upper and lower levels, which results in a parent–child relationship between coaches and athletes (benevolent coaching style). While several studies have attempted to incorporate coaches’ helping and hindering behaviors (Rocchi et al., 2017; Pulido et al., 2018), these efforts have not focused on China. Thus, this study added the benevolent coaching style dimension to consider cultural variations. Relatedness includes the need to connect with others and desire to experience and receive love and care (Deci and Ryan, 2000). This study integrated the coaching style of Chinese coaches, broadening the coach-athlete relatedness need within the SDT, particularly in the context of the distinct superior-subordinate relationships between Chinese coaches and athletes.

### 5.1 Limitations and future research directions

The three dimensions in this scale were created based on existing scales (Farh et al., 2000; Reinboth et al., 2004; Zhao and Zhou, 2022). The items were not directly drawn from the interview data, which may have resulted in a limited understanding of the coaching behaviors employed by Chinese coaches. To address this issue, future research should incorporate expert interviews with Chinese coaches and players. By generating localized items for the three factors relevant to the Chinese context, this method would refine the dimensions of Chinese coaches’ styles. The circumplex model, which includes four types (autonomy support, control, structure, and chaos), has recently been used to characterize coaches’ (de)motivating practices in a more thorough and nuanced way (Delrue et al., 2019). Not all types were incorporated into the Chinese coaching styles in this study due to the lack of appropriate supporting data. Future research should concentrate on including more pertinent coaching styles based on the circumplex model, thus capturing a wider variety of Chinese coaches’ behaviors.

## 6 Conclusion

This study found that (1) the benevolent coaching style occupied a significant explanatory weight in the Chinese cultural context; (2) the controlling (negative conditioned regard and excessive personal control) and autonomy-supportive coaching styles were culturally compatible with both Eastern and Western athletes; and (3) the benevolent and autonomy-supportive coaching styles had a positive impact on athletes, whereas the controlling coaching style had a negative impact. This study demonstrated that benevolence, exhibited in the coaches’ parental care for their athletes, is an important coaching style in China, in addition to autonomy-supportive and controlling coaching styles. In light of previous research, this study developed the CCISS. To establish the distinctive cultural characteristics of benevolence, more research should be conducted on how benevolence affects athletes from Western cultures.

## Data availability statement

The original contributions presented in the study are included in the article/supplementary material, further inquiries can be directed to the corresponding author.

## Ethics statement

The studies involving humans were approved by Human Experimental Ethics Inspection of Guangzhou Sport University. The studies were conducted in accordance with the local legislation and institutional requirements. Written informed consent for participation in this study was provided by the participants’ legal guardians/next of kin.

## Author contributions

WS: Validation, Writing – original draft. DZ: Conceptualization, Funding acquisition, Writing – review & editing.
